# Editorial: Neuromechanisms underlying motor imagery training (MIT) and roles of MIT in motor skill acquisition and muscle strength enhancement in both sport and rehabilitation settings

**DOI:** 10.3389/fpsyg.2023.1344889

**Published:** 2023-12-13

**Authors:** Wan X. Yao, William Land, Guang H. Yue

**Affiliations:** ^1^Department of Kinesiology, College for Health, Community, and Policy, The University of Texas at San Antonio, San Antonio, TX, United States; ^2^Center for Mobility and Rehabilitation Engineering Research, Kessler Foundation, East Hanover, NJ, United States; ^3^Rutgers New Jersey Medical School, Rutgers, The State University of New Jersey, Newark, NJ, United States

**Keywords:** motor imagery training, mental practice, motor skill acquisition, muscle strength enhancement, cognitive neuroscience, aging

Over the past decades, motor imagery training (MIT) has risen as a compelling strategy for amplifying motor skills and muscle strength, making noteworthy contributions to both sports performance and rehabilitation settings (Toth et al., 2020). This editorial seeks to shed light on a series of five recent articles featured in a Frontiers Research Topic. The objective of this Frontiers Research Topic was to offer a thorough examination of the neuromechanisms that form the foundation of MIT and to delineate its multifaceted roles in the realms of motor skill acquisition and muscle strength enhancement.

MIT, also known as mental practice or mental rehearsal, represents a dynamic paradigm in the field of motor learning and rehabilitation. At its core, MIT involves the mental rehearsal of a physical action without engaging in the overt physical movements associated with the action (Decety, 1996; Krüger et al., 2020). This cognitive process engages neural networks associated with motor planning and execution, offering a unique avenue to enhance motor skills, optimize performance, and facilitate rehabilitation across diverse populations.

MIT can be executed using two primary forms of mental imagery—internal and external imagery. Internal motor imagery (IMI), also known as kinesthetic or first-person imagery, requires an individual to envision or mentally simulate the experience of performing an exercise from within the body. This involves adopting a first-person perspective to create a vivid mental representation of the activity. In contrast, external motor imagery (EMI), alternatively known as third-person visual imagery, involves an individual seeing or visualizing themselves performing a task from an external standpoint—similar to observing oneself in a mirror executing an exercise, adopting a third-person perspective (Yao et al., 2013). This cognitive simulation activates similar neural pathways as those engaged during actual physical performance, creating a bridge between thought and action (Hétu et al., 2013). Consequently, MIT is rooted in the understanding that the brain processes imagined and executed movements through overlapping neural circuits. When an individual mentally rehearses a movement, the brain elicits patterns of neural activity comparable to those observed during the physical execution of the same movement. This underscores MIT's profound implications for skill acquisition, performance enhancement, and rehabilitation in various contexts, including sports, clinical settings, and beyond.

In the following articles within this Frontiers Research Topic, the intricacies of MIT are explored through a combination of original studies and reviews, encompassing systematic reviews and meta-analyses. These contributions delve into diverse facets, including its applicability in children, age-specific benefits, bilateral transfer effects, neurophysiological underpinnings, and the integration of movement variability. Collectively, these studies significantly enhance our understanding of MIT, playing a pivotal role in its ongoing evolution as a potent tool for motor learning, performance optimization, and rehabilitation. For instance, Saleem's mini-review delves into critical nuances of MIT in children, shedding light on its applicability and the challenges associated with measuring it in developmental contexts. Saleem's work not only examines past and current research describing motor imagery ability in children from the theoretical, developmental, and neurological lens but also systematically analyzes the properties of three widely used operations—the movement imagery questionnaire in children (MIQ-C), the Florida praxis imaginary questionnaire (FPIQ-C), and the mental chronometry paradigm (MCP)—to measure MI and its dimensions in children. Additionally, it advocates for early intervention strategies and the optimization of skill acquisition.

Surprisingly and intriguingly, Yao et al., in their systematic review and meta-analysis (Liu et al.), reveal that the elderly may experience greater muscle strength benefits from Motor Imagery Training (MIT) than young adults. Notably, their findings indicate that smaller muscle groups, such as finger muscles, derive more substantial benefits from MIT compared to larger muscle groups like arm and leg muscles. Furthermore, their study (Liu et al.) demonstrates that IMI training is more effective than EMI training in improving muscle strength. These discoveries carry significant implications for the design of targeted rehabilitation programs tailored to meet the specific needs of the aging population. In a separate systematic review and meta-analysis study, Yao et al. explore the concept of bilateral transfer in motor performance following MIT. Unpacking the intricate relationship between motor imagery and performance, this work provides a nuanced understanding of how MIT facilitates bilateral transfer, impacting both motor skill acquisition and muscle strength enhancement across various contexts, further amplifying its potential applications in both sports and rehabilitation.

Lajtos et al. extend the exploration into the neurophysiological aspects of MIT by investigating the effects of handedness on brain oscillatory activity during imagery and execution of hand movements. Notably, reveals that the right-handed group tended to exhibit more bilateral patterns than the left-handed group, contrary to earlier research results. This finding paves the way for tailored interventions that consider individual differences in implementing MIT and understanding bilateral transfer in motor skill acquisition. Additionally, the research indicates a stronger activation during motor imagery compared to motor execution in both right-handed and left-handed groups. Addressing a crucial yet often overlooked aspect of MIT, Lindsay et al. focus on movement variability. Their work underscores the importance of incorporating variability into imagery training programs, marking a paradigm shift in how MIT is approached in both sporting and rehabilitative contexts. This consideration opens new avenues for refining training protocols, with potential benefits of implementing MIT for enhancing adaptability and resilience in motor performance, spanning both sports and rehabilitation contexts. Furthermore, the research suggests that practitioners should view MIT as a low-risk strategy for incorporating movement variability into the rehabilitation process and cultivating adaptable movement skills.

In conclusion, this Frontiers Research Topic contributes significantly to the intricate tapestry of knowledge, advancing our comprehension of the neuromechanisms underlying Motor Imagery Training (MIT) and its diverse roles in motor skill acquisition and muscle strength enhancement. As MIT continues its evolution, bridging the gap between research and application, these studies lay the foundation for more effective and tailored interventions in both sports and rehabilitation settings. The compilation of studies in this Research Topic suggests that future research should focus on exploring individual differences in response to MIT, taking into account factors such as cognitive abilities, personality traits, and learning styles. Developing personalized MIT protocols to optimize effectiveness based on these individual characteristics, also including attention, motivation, and emotional states (Wulf and Lewthwaite, 2016), can further enhance the outcomes of MIT. Additionally, future studies are essential to explore the integration of technology, such as virtual reality, augmented feedback, or mobile applications, to improve the delivery and accessibility of MIT interventions. This forward-looking approach will contribute to the continued growth and refinement of MIT as a valuable tool in enhancing motor performance and rehabilitation outcomes.

## Author contributions

WY: Conceptualization, Investigation, Project administration, Writing – original draft, Writing – review & editing. WL: Conceptualization, Writing – review & editing. GY: Conceptualization, Writing – original draft, Writing – review & editing.
